# LncRNA nonnmmut065573 promotes post-myocardial infarction cardiac fibrosis and activates the TGF-β1/ZEB1 pathway

**DOI:** 10.1186/s13619-025-00275-5

**Published:** 2026-01-21

**Authors:** Chaowei Hu, Lijie Han, Zhiyong Du, Huahui Yu, Yunhui Du, Linyi Li, Haili Sun, Yu Wang, Xiaoqian Gao, Xuechun Sun, Zihan Zhang, Lanqing Liu, Yanjing Zhang, Yanwen Qin

**Affiliations:** Key Laboratory of Remodeling-Related Cardiovascular Diseases, Beijing Anzhen Hospital, Capital Medical University, Beijing Institute of Heart, Lung and Blood Vessel Diseases, Beijing, 100029 China

**Keywords:** LncRNA-IH, Myocardial infarction, Cardiac fibroblasts, TGF-β1 signaling pathway, ZEB1

## Abstract

Cardiac fibrosis following myocardial infarction (MI) is a critical determinant of progressive cardiac dysfunction, yet the underlying mechanisms driving this pathological process remain incompletely understood. Elucidating these regulatory pathways holds profound implications for improving post-MI prognosis.

Our prior work demonstrated that chronic intermittent hypoxia (CIH) exacerbates cardiac fibrosis while modulating the expression of long non-coding RNA (lncRNA) nonnmmut065573 (tentatively designated LncRNA-IH) in cardiac tissues. Herein, we sought to determine the role of LncRNA-IH in post-MI cardiac fibrosis and its underlying mechanisms. Using a C57BL/6 mouse model of MI, we established a mouse model with cardiac-specific overexpression of LncRNA-IH to evaluate post-MI cardiac fibrosis. In vitro, primary cardiac fibroblasts (MCF) and the PA12 cell line were subjected to LncRNA-IH overexpression or siRNA-mediated knockdown, and cell proliferation and migration were assessed. Transcriptomic profiling was performed to characterize LncRNA-IH-induced changes in cardiac gene expression and signaling pathways, aiming to elucidate the molecular mechanisms involved.

Results showed that CIH significantly exacerbated post-MI cardiac fibrosis, and LncRNA-IH was predominantly localized to cardiac fibroblasts. Cardiac-specific overexpression of LncRNA-IH in MI mice markedly exacerbated post-MI cardiac dysfunction and fibrosis. In vitro, LncRNA-IH overexpression significantly enhanced the proliferation and migration capacities of primary cardiac fibroblasts and PA12 cells, whereas these effects were abrogated by LncRNA-IH knockdown. Transcriptomic analysis revealed that LncRNA-IH elicited significant alterations in cardiac gene expression profiles, specifically activating the TGF-β1 signaling pathway and upregulating the expression of its downstream target, ZEB1.

Collectively, our findings indicate that LncRNA-IH promotes cardiac fibroblast proliferation and migration, thereby exacerbating post-MI cardiac remodeling, at least in part through activation of the TGF-β1 signaling pathway. This study identifies LncRNA-IH as a potential therapeutic target for mitigating post-MI cardiac fibrosis and preserving cardiac function.

**Supplementary Information:**

The online version contains supplementary material available at 10.1186/s13619-025-00275-5.

## Background

Myocardial infarction (MI) remains a leading cause of mortality and morbidity globally, with post-MI cardiac fibrosis emerging as a pivotal driver of progressive heart failure (Benjamin et al. 2019). Cardiac fibrosis, characterized by excessive extracellular matrix (ECM) deposition and cardiac fibroblast activation, disrupts myocardial structure and impairs contractile function, ultimately leading to irreversible cardiac remodeling (Travers et al. 2016; Frangogiannis et al. 2019). Despite advances in revascularization therapies, the clinical management of post-MI fibrosis remains limited, underscoring the need to identify novel regulatory targets (Ponikowski et al. 2016).

Long non-coding RNAs (lncRNAs) have emerged as key modulators of cardiac pathophysiology, with accumulating evidence linking their dysregulation to fibrosis, hypertrophy, and heart failure (Statello et al. 2021; Dong et al. 2022; Wang et al. 2021). LncRNAs exert regulatory effects through diverse mechanisms, including transcriptional modulation and signaling pathway activation, making them promising therapeutic candidates (Sun et al. 2025; Wen et al. 2024; Xie et al. 2025). Our prior work identified LncRNA-IH (nonnmmut065573) as a hypoxia-responsive lncRNA in cardiac tissues, with chronic intermittent hypoxia (CIH)—a common comorbidity in sleep-disordered breathing—exacerbating post-MI fibrosis while upregulating LncRNA-IH (Hu et al. 2021).

CIH is known to exacerbate post-MI cardiac dysfunction by promoting oxidative stress and inflammatory responses, but its interaction with lncRNA-mediated fibrosis remains underexplored (Cain et al. 2015). Cardiac fibroblasts, the primary effector cells in fibrosis, undergo activation, proliferation, and migration post-MI, processes tightly regulated by signaling pathways such as TGF-β1 (Nagaraju et al. 2017). The TGF-β1 pathway, a master regulator of fibrosis, drives myofibroblast differentiation and ECM synthesis via downstream targets like ZEB1, a transcription factor linked to epithelial-mesenchymal transition and ECM remodeling (Lamouille et al. 2014; Meng et al. 2016; Zhan and Kanwar 2014). However, whether lncRNAs bridge CIH and TGF-β1/ZEB1 activation in post-MI fibrosis is unclear.

Herein, we hypothesized that LncRNA-IH promotes post-MI cardiac fibrosis by activating cardiac fibroblasts via the TGF-β1/ZEB1 pathway. Using in vivo MI models with LncRNA-IH overexpression and in vitro fibroblast assays, we investigated its role in fibrosis and remodeling. Transcriptomic profiling further elucidated underlying molecular mechanisms. This study aims to identify LncRNA-IH as a novel therapeutic target for post-MI fibrosis.

## Results

### LncRNA-IH is upregulated by intermittent hypoxia in cardiac fibroblasts

Our previous studies demonstrated that chronic intermittent hypoxia (CIH) exacerbates post-myocardial infarction (post-MI) cardiac fibrosis while modulating the expression of cardiac long non-coding RNAs (lncRNAs) (Hu et al. 2021). To investigate whether LncRNA-IH is regulated by intermittent hypoxia in the setting of MI, we established a mouse model of MI and exposed the mice to intermittent hypoxia. Consistent with our prior findings, Masson’s trichrome staining of cardiac sections revealed that CIH exacerbated post-MI cardiac fibrosis (Fig. S1A). Fluorescence in situ hybridization (FISH) staining demonstrated that MI increased cardiac LncRNA-IH expression, with a further upregulation observed under intermittent hypoxia (Fig. S1B); these findings were corroborated by quantitative real-time polymerase chain reaction (qPCR) results, which confirmed that CIH promoted LncRNA-IH expression in MI hearts (Fig. S1C). To explore whether LncRNA-IH contributes to post-MI cardiac fibrosis, we co-stained LncRNA-IH with the cardiac fibroblast marker col1A2. Merged images showed that LncRNA-IH was predominantly expressed in cardiac fibroblasts in post-MI hearts (Fig. S1D, S2).

To further confirm that CIH upregulates LncRNA-IH expression in cardiac fibroblasts, we isolated primary cardiac fibroblasts (MCF) from mouse hearts and maintained the PA12 cell line through continuous subculture. Both fibroblast types were exposed to CIH for 72 h, and results showed that CIH significantly increased LncRNA-IH expression in these fibroblasts (Fig. S1E). Collectively, these results indicate that LncRNA-IH is expressed in cardiac fibroblasts and is regulated by CIH during post-MI cardiac fibrosis, suggesting a potential profibrotic role in this pathological process.

### LncRNA-IH overexpression exacerbates post-mi cardiac remodeling and dysfunction in mice

**Fig. 1:**
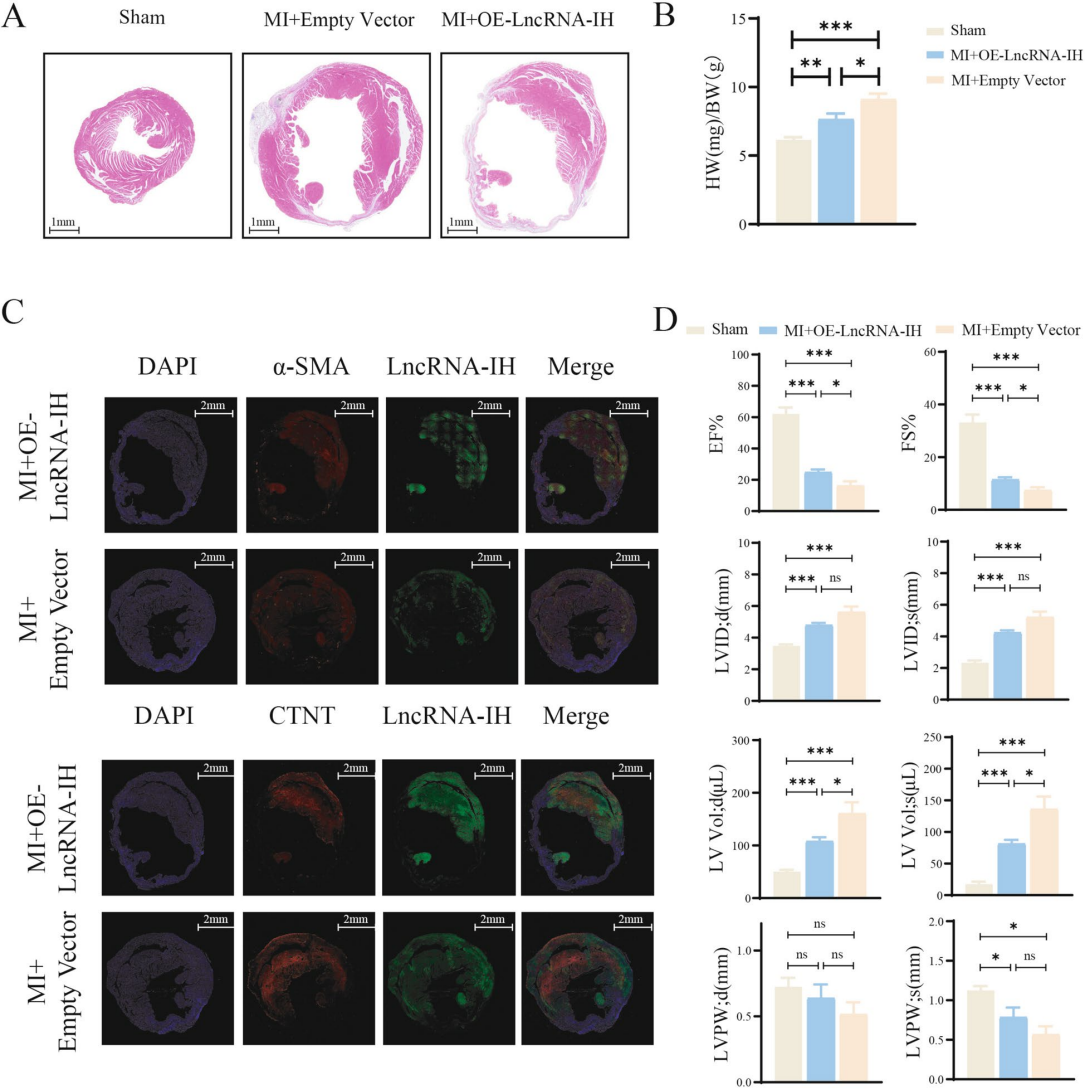
LncRNA-IH overexpression exacerbates post-MI cardiac remodeling and dysfunction in mice. **A** Cross sections of heart tissues were stained by HE and cardiomyocyte inflammation was determined. *n* = 6 for each group. **B** HW/BW indicates the heart weight/body weight. *n* = 7 for Sham, *n* = 6 for MI + OE-LncRNA-IH and *n* = 6 for MI + Empty Vector. *P < 0.05, **P < 0.01, ***P < 0.001. Data are presented as mean ± SEM. **C** Representative images of fluorescent in situ hybridizations (FISH) of LncRNA-IH co-labeled with DAPI (blue) and α-SMA (myofibroblasts) (red) or CTNT (cardiomyocytes) (red) to observe the localization of LncRNA-IH in cardiac cells. **D** Quantification of EF, FS, LV Vol-d (left ventricular diastolic volume), LV Vol-s (left ventricular systolic volume), LVIDd (left ventricular internal diastolic diameter), LVIDs (left ventricular internal systolic diameter), LVPWd (left ventricular diastolic posterior wall), LVPWs (left ventricular systolic posterior wall). *n* = 7 for Sham, *n* = 8 for MI + OE-LncRNA-IH and *n* = 6 for MI + Empty Vector. *P < 0.05, **P < 0.01, ***P < 0.001, ns, non-significant. Data are presented as mean ± SEM

To investigate the role of LncRNA-IH in the post-myocardial infarction (MI) heart of mice, we constructed an adeno-associated virus for LncRNA-IH. Two weeks after MI surgery, the virus was injected into mice via the tail vein, with an empty virus control group set up simultaneously. Four weeks later, cardiac HE staining results (Fig. 1A) showed that cardiac overexpression of LncRNA-IH could increase the left ventricular cavity volume and enhance ventricular wall thickness. Compared with the sham operation group, MI significantly increased the heart weight-to-body weight ratio (HW/BW) of mice, and overexpression of LncRNA-IH aggravated this phenomenon (Fig. 1B). Echocardiographic and related analyses further revealed that MI-induced cardiac dysfunction was more severe in the LncRNA-IH overexpression group, as evidenced by decreased left ventricular ejection fraction (LVEF), fractional shortening (FS), and changes in left ventricular internal diameter (LVID) (Fig. 1C). Collectively, these findings indicate that overexpression of LncRNA-IH exacerbates post-MI cardiac structural remodeling (characterized by enlarged left ventricular cavity, increased ventricular wall thickness, and aggravated cardiac hypertrophy reflected by elevated HW/BW) and functional impairment in mice, suggesting that LncRNA-IH may act as a pathogenic factor in the progression of cardiac damage after myocardial infarction.

### Enhanced LncRNA-IH expression aggravates myocardial fibrosis in post-MI mice

**Fig. 2:**
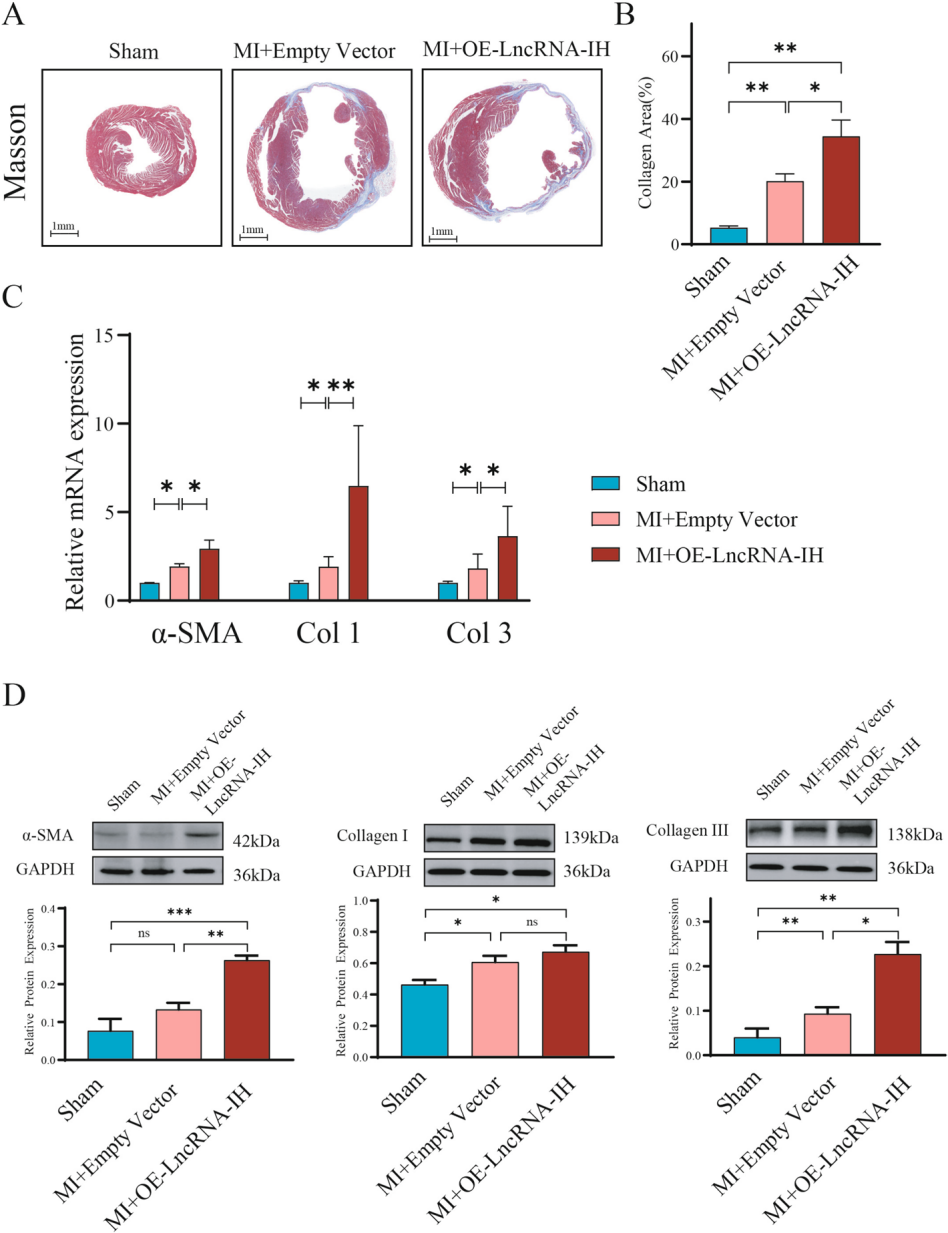
LncRNA-IH promotes cardiac fibrosis after myocardial infarction. **A**-**B** Cross sections of heart tissues were stained by Masson, and cardiac fibrosis were determined. *n* = 6 for each group. **C** The expression of α-SMA, Col1 and Col3 were validated by qPCR analysis. *n* = 3/group. *P < 0.05. Data are presented as mean ± SEM. **D** Levels of α-SMA, Col1 and Col3 in cardiac tissues were detected by Western blotting. The relative intensities are shown in the panel. GAPDH served as a loading control. *n* = 6 per group. Data are presented as *P < 0.05, **P < 0.01, ***P < 0.001. Data are presented as mean ± SEM

Myocardial fibrosis, a critical pathological feature of post-infarction cardiac remodeling, was further evaluated to explore the role of LncRNA-IH. Masson staining (Fig. 2A) results (consistent with the trend in collagen area shown in Fig. 2B) demonstrated that overexpression of LncRNA-IH significantly promoted cardiac fibrosis: compared with the MI group, the collagen-positive area was markedly enlarged in the MI + OE-LncRNA-IH group, indicating more severe extracellular matrix (ECM) deposition.

To validate the molecular basis of this fibrotic response, we detected the expression of key fibrosis-related markers at both transcriptional and translational levels. Quantitative real-time PCR (qPCR) analysis revealed that, relative to the MI group, the mRNA levels of α-smooth muscle actin (α-SMA, a marker of myofibroblast activation), collagen type I (Col1), and collagen type III (Col3)—core components of ECM—were significantly upregulated in the MI + OE-LncRNA-IH group (Fig. 2C). These transcriptional changes were further confirmed at the protein level: Western blot results showed that the protein expression of α-SMA, Col1, and Col3 was also significantly increased in the LncRNA-IH overexpression group compared with the MI group (Fig. 2D). Collectively, these findings indicate that LncRNA-IH overexpression exacerbates post-MI myocardial fibrosis, which is associated with enhanced activation of myofibroblasts and increased synthesis of ECM components (Col1 and Col3).

### LncRNA-IH promotes cardiac fibroblast proliferation, migration, and fibrotic activation in vitro

To explore the direct regulatory effects of LncRNA-IH on cardiac fibroblasts and the underlying mechanisms of its pro-fibrotic role, we performed in vitro experiments using primary cardiac fibroblasts (MCF) and the fibroblast cell line PA12. Functional assays revealed that LncRNA-IH overexpression significantly enhanced primary cardiac fibroblast proliferation, as evidenced by increased BrdU-positive cells in the OE-LncRNA-IH group compared to the control group (Fig. S3A). Consistently, Transwell migration assays demonstrated a marked increase in migrated primary cardiac fibroblasts following LncRNA-IH overexpression, indicating its ability to promote cell migration (corresponding to migration-related results, Fig. S3B). At the molecular level, Western blot analysis confirmed that overexpression of LncRNA-IH significantly upregulated the protein expression of α-SMA—a key marker of fibroblast-to-myofibroblast differentiation—in primary cardiac fibroblasts (Fig. S3C).

**Fig. 3:**
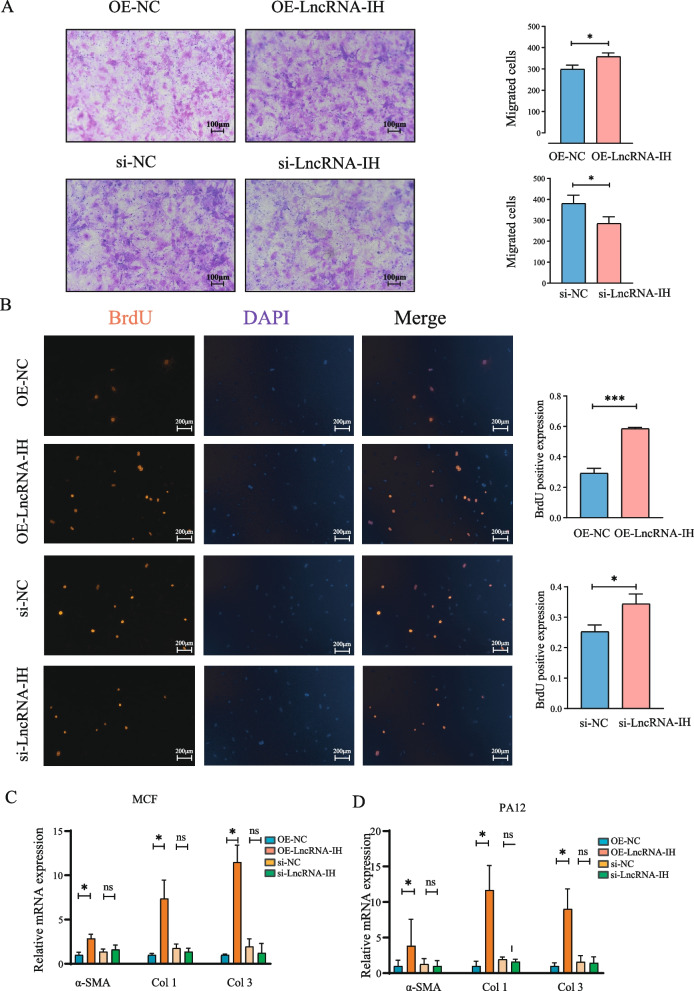
LncRNA-IH promotes the proliferation and migration of cardiac fibroblasts. **A** Transwell assay to determine the effect of LncRNA-IH on the migration of cardiac fibroblasts. Data are presented as Mean ± SEM, with *n* = 3/group. *P < 0.05 and **P < 0.01 vs. the NC group. **B** Quantitative analysis of the percentage of BrdU-positive cells in each group was performed, and the data are presented as Mean ± SEM, with *n* = 3/group. One-way analysis of variance (One-way ANOVA) and Tukey’s multiple comparison test were used. *P < 0.05 and **P < 0.01 vs. the NC group. **C, D** The expression of α-SMA, Col1 and Col3 were validated by qPCR analysis in fibroblasts in vitro. *n* = 3/group. *P < 0.05. Data are presented as mean ± SEM

To validate the specificity of LncRNA-IH’s effects, we knocked down LncRNA-IH using siRNA in both MCF and PA12 cells. Quantitative real-time PCR (qPCR) results showed that transfection with si-LncRNA-IH significantly reduced LncRNA-IH expression in both cell types compared to the si-NC control (Fig. S4), confirming efficient silencing. Functional reciprocity was observed: while LncRNA-IH overexpression promoted fibroblast migration, its knockdown exerted the opposite effect, suppressing migration (Fig. 3A). Similarly, proliferation assays (consistent with BrdU staining trends in Fig. 3B) revealed that LncRNA-IH overexpression enhanced fibroblast proliferation, whereas knockdown inhibited this process. Moreover, qPCR analysis of fibrotic markers showed that LncRNA-IH overexpression significantly increased the mRNA levels of α-SMA, Col1, and Col3 in both MCF and PA12 cells. Conversely, siRNA-mediated LncRNA-IH knockdown reversed these effects, leading to downregulated mRNA expression of these markers (Fig. 3C, D).

Collectively, these in vitro findings demonstrate that LncRNA-IH directly promotes cardiac fibroblast proliferation, migration, and fibrotic activation (as indicated by upregulated α-SMA, Col1, and Col3), with these effects being reversible upon LncRNA-IH silencing. This further supports that LncRNA-IH acts as a critical driver of cardiac fibrosis through regulation of fibroblast function.

### Transcriptomic profiling identifies LncRNA-IH-regulated candidate genes associated with myocardial fibrosis

To explore the molecular mechanisms underlying LncRNA-IH-mediated promotion of cardiac fibroblast proliferation and migration, we performed transcriptomic analysis on cardiac tissues from Sham, MI, and MI + LncRNA-IH (MI-LncRNA-IH) groups.

**Fig. 4:**
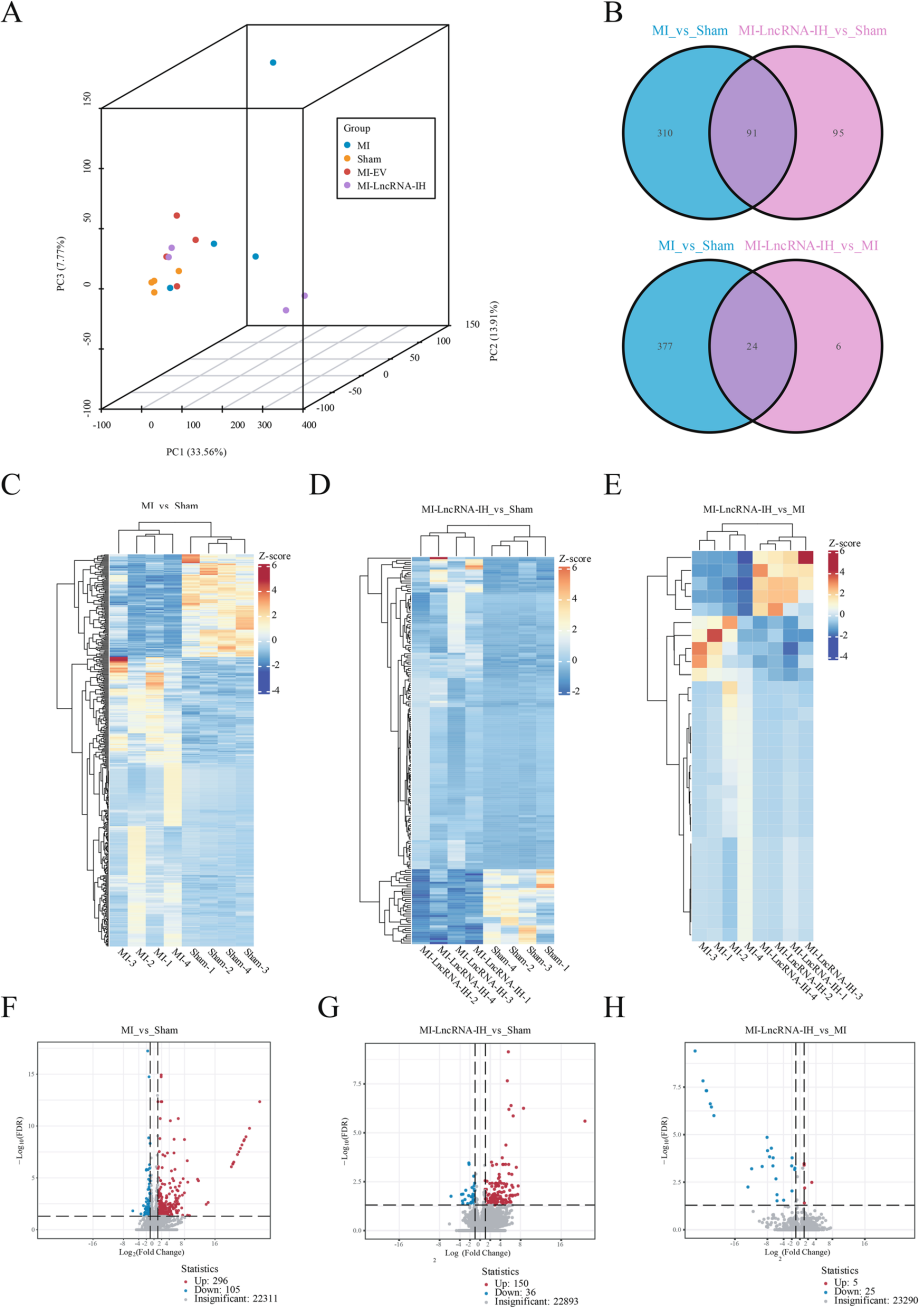
Specific mRNAs regulated by LncRNA-IH. **A** A 3D PCA plot of samples from different groups (MI, Sham, MI-EV, MI-LncRNA-IH), where PC1 explains 33.56% of the variance. The plot shows the distribution characteristics of samples in each group (MI group *n* = 4, Sham group *n* = 4, MI-EV group *n* = 4, MI-LncRNA-IH group *n* = 4) in the principal component space. **B** Venn diagram data of differentially expressed mRNAs between “MI vs Sham”, “MI-LncRNA-IH vs Sham” and “MI-LncRNA-IH vs MI”, showing gene expression changes between the intervention group and the Sham group. **C**-**E** Heat map depicting the changes in mRNAs in MI Vs Sham group, MI-LncRNA-IH Vs Sham group and MI-LncRNA-IH Vs MI group. **F**–**H** Volcano plots of the significantly different mRNAs between MI Vs Sham group, MI-LncRNA-IH Vs Sham group and MI-LncRNA-IH Vs MI group

Beta-diversity analysis via principal component analysis (PCA) revealed distinct clustering patterns among the three groups, with PC1 (33.56%) and PC2 (13.91%) collectively explaining the major variance, indicating significant transcriptional differences induced by MI and LncRNA-IH overexpression (Fig. 4A). Differential gene expression analysis identified 401 genes with significant changes in the MI vs. Sham comparison, while 186 genes were differentially expressed in the MI-LncRNA-IH vs. Sham group. A Venn intersection of these two gene sets revealed 91 overlapping differentially expressed genes (DEGs), representing core transcriptional responses common to both MI injury and LncRNA-IH overexpression relative to the normal state (Fig. 4B). Further, 30 DEGs were identified in the MI-LncRNA-IH vs. MI comparison, and intersection with the 401 MI vs. Sham DEGs yielded 24 shared genes—these genes are likely regulated by LncRNA-IH and involved in MI-induced pathological processes (Fig. 4B). These 24 candidate genes included *Bahcc1*, *Sftpa1*, *Cldn18*, *Nkx2-1*, *Tnnt3*, *Scnn1g*, *Sfta3ps*, *Gabrp*, *Zfp608*, *Mybpc1*, *Myh1*, *Grip2*, *Myh2*, *Mylpf*, *Ryr1*, *Tnni2*, *Pvalb*, *Actn3*, *Resf1*, *Atp2a1*, *Myh4*, *Myoz1*, *Ctxn3*, and *Spon2*. While most of these genes have not been directly linked to cardiac fibrosis in previous studies, their consistent dysregulation in MI and LncRNA-IH-overexpressing contexts suggests potential roles in fibrotic remodeling—for instance, *Spon2* has been implicated in extracellular matrix (ECM) remodeling and cell migration in other tissues, and *Ctxn3* may modulate cell adhesion, a process relevant to fibroblast activation (Jin et al. 2024).

Hierarchical clustering heatmaps (Fig. 4C–E) visualized the expression patterns of DEGs in each comparison, confirming robust transcriptional divergence: MI vs. Sham showed widespread gene expression changes, while MI-LncRNA-IH vs. MI displayed more focused alterations, aligning with the smaller number of DEGs. Volcano plots (Fig. 4F–H) further illustrated the distribution of DEGs, with clear separation between upregulated and downregulated genes, validating the statistical significance of these transcriptional changes. Collectively, these transcriptomic data highlight 24 LncRNA-IH-regulated genes that are co-dysregulated in MI, providing potential molecular targets for understanding how LncRNA-IH exacerbates cardiac fibrosis through modulation of fibroblast function.

### GO functional enrichment analysis of DEGs and relevance to cardiac fibrosis

**Fig. 5:**
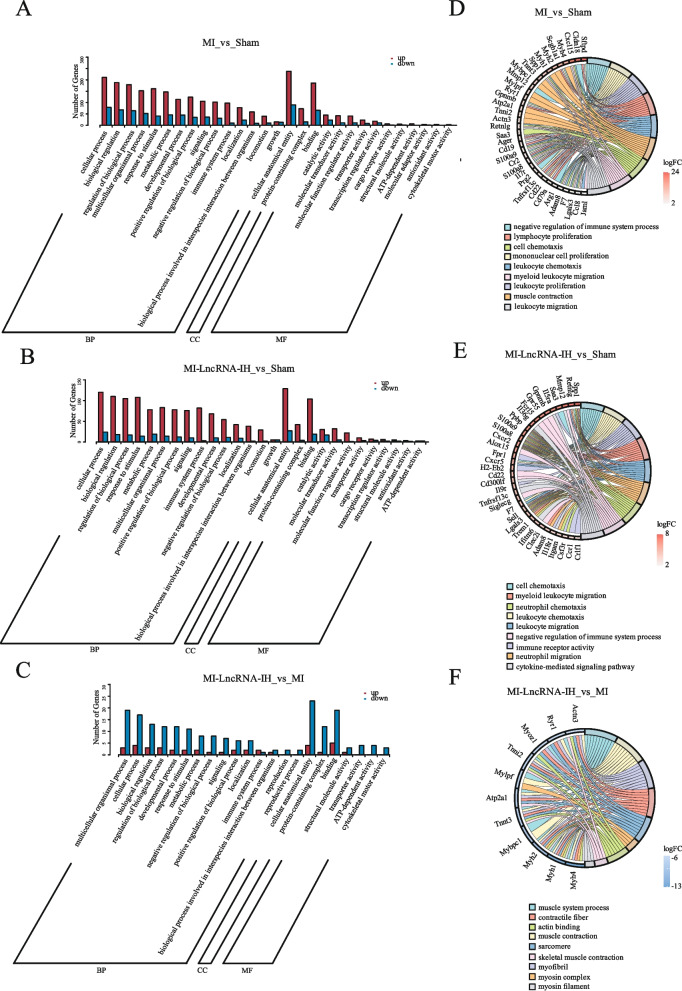
GO functional annotation analysis of LncRNA-IH-associated mRNAs after MI. **A**-**C** Data show bioinformatics analysis of the GO terms in biological process, cellular component and molecular function of RNAs enriched in MI or MI-LncRNA-IH as compared to Sham and MI-LncRNA-IH vs MI **D**-**F** The GO enrichment chord plots illustrate the enrichment profiles of relevant genes in different comparison groups (MI-LncRNA-IH vs MI, MI-LncRNA-IH vs Sham, and MI vs Sham), involving biological functions such as muscle system processes and immune-related processes along with corresponding logFC values

Gene Ontology (GO) enrichment analysis was performed to explore the functional relevance of differentially expressed genes (DEGs) in the context of LncRNA-IH-mediated effects. In the Biological Process (BP) category, the MI vs. Sham and MI-LncRNA-IH vs. Sham groups showed overlapping enrichment, with DEGs primarily concentrated in cellular process, biological regulation, and regulation of biological process (Fig. 5A–C). Among these, cellular process—which encompasses fibroblast proliferation, migration, and myofibroblast differentiation—was most relevant to cardiac fibrosis, as these cellular activities are core to fibrotic initiation and progression. The MI-LncRNA-IH vs. MI group, reflecting LncRNA-IH-specific effects, displayed the highest DEG enrichment in multicellular organismal process, cellular process, and biological regulation (Fig. 5C).

For the Cellular Component (CC) category, all three groups showed consistent enrichment of DEGs in protein-containing complex and cellular anatomical entity (Fig. 5A–C). In the Molecular Function (MF) category, the MI vs. Sham and MI-LncRNA-IH vs. Sham groups shared enrichment in binding, catalytic activity, and molecular transducer activity (Fig. 5A, B), while the MI-LncRNA-IH vs. MI group was most enriched in binding and structural molecule activity (Fig. 5C); notably, binding mediates key fibrotic interactions (e.g., TGF-β receptor binding), and structural molecule activity is linked to excessive extracellular matrix (ECM) deposition, a hallmark of fibrosis.

Correlation analysis of DEGs with functional terms (Fig. 5D–F) and bubble plots of enriched pathways (Fig. S5A–C) further validated fibrotic relevance. Terms related to ECM organization and cell migration (implicit in cellular process) were associated with DEGs in all groups, reflecting core fibrotic events like ECM remodeling and fibroblast recruitment. In the MI-LncRNA-IH vs. MI group, enrichment of structural molecule activity (Fig. S5C) aligned with ECM component upregulation, while binding (Fig. S5A–B) underscored enhanced signaling for collagen synthesis. Collectively, these results indicate that LncRNA-IH modulates fibrosis through cellular process (regulating fibroblast behavior) and structural molecule activity (promoting ECM deposition), with binding as a key molecular driver.

Gene Set Enrichment Analysis (GSEA) was performed to explore the biological functions and pathways associated with LncRNA-IH in three comparison groups: MI-LncRNA-IH vs Sham, MI vs Sham, and MI-LncRNA-IH vs MI (Fig. S6). The results revealed enrichment of multiple GO functions, including endopeptidase regulator activity, peptidase regulator activity, negative regulation of immune system process, and leukocyte proliferation, as well as several KEGG pathways such as Cell adhesion molecules, Leukocyte transendothelial migration, and Oxidative phosphorylation. These findings suggest that LncRNA-IH may be involved in regulating processes related to immune response, cell adhesion, and energy metabolism in the context of myocardial infarction.

### LncRNA-IH may promote cardiac fibrosis via the TGF-β1 signaling pathway

**Fig. 6:**
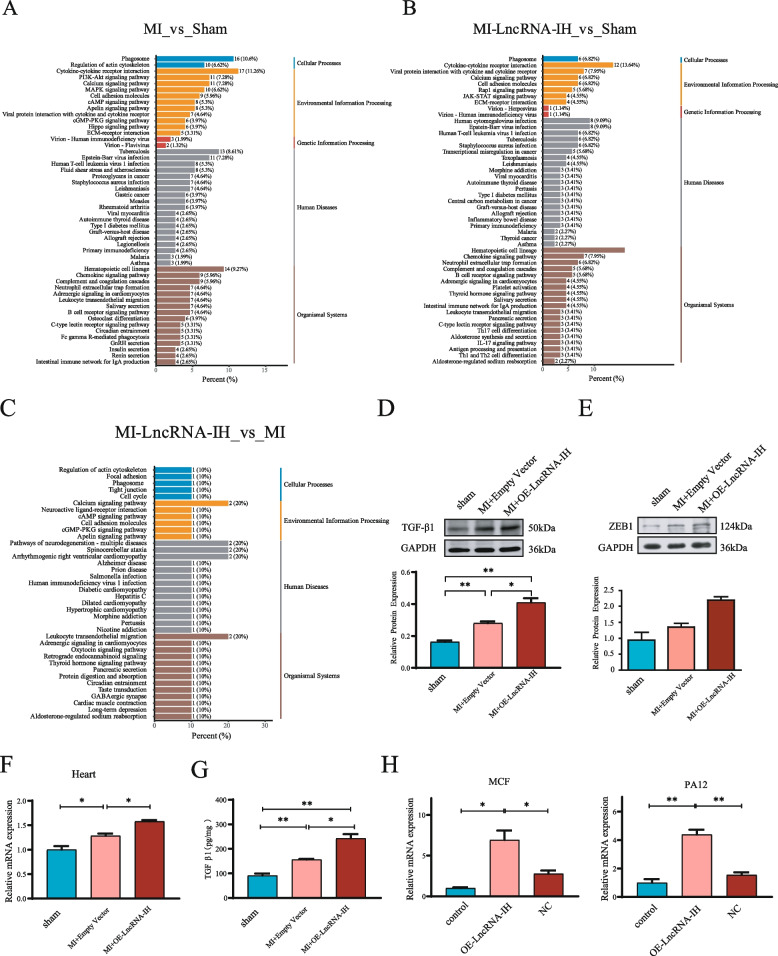
LncRNA-IH activates the TGFβ−1 signaling pathway after myocardial infarction. **A**-**C** KEGG enrichment bar plots display the significantly enriched signaling pathways of differentially expressed genes in MI vs Sham, MI-LncRNA-IH vs MI and MI-LncRNA-IH vs Sham groups. The x-axis indicates the percentage of each pathway, with different colors representing functional classes of pathways (e.g., Cellular Processes, Human Diseases). Numbers adjacent to pathway names denote the gene count and proportion of genes in each pathway, respectively. **D** Levels of TGF-β1 in cardiac tissues were detected by Western blotting in different groups. The relative intensities are shown in the panel. GAPDH served as a loading control. *n* = 6 per group. Data are presented as *P < 0.05, **P < 0.01. Data are presented as mean ± SEM. **E** Protein and mRNA levels of ZEB1 in cardiac tissues were detected in different groups. The relative intensities are shown in the panel. *n* = 6 per group. Data are presented as *P < 0.05. Data are presented as mean ± SEM. **F** ELISA results demonstrate the relative expression levels of TGF-β1 in the Sham, MI, and MI + OE-LncRNA-IH groups. Data are presented as mean ± standard error of the mean (SEM), with *P < 0.05 and **P < 0.01 indicating statistically significant differences between groups. **G**-**H** The mRNA expression levels of ZEB1 were determined in distinct groups within the MCF and PA12 cell lines, respectively. *n* = 6 per group. Data are presented as mean ± standard error of the mean (SEM), with *P < 0.05 denoting statistically significant differences

KEGG pathway enrichment analysis of differentially expressed genes across groups (Fig. 6A–C) revealed that key enriched pathways in the MI vs. Sham, MI-LncRNA-IH vs. Sham, and MI-LncRNA-IH vs. MI comparisons included ECM-receptor interaction, Calcium signaling pathway, Rap1 signaling pathway, and Cytokine-cytokine receptor interaction. These pathways are closely associated with the TGF-β1 signaling pathway, as they participate in downstream processes of TGF-β1, such as extracellular matrix (ECM) deposition and cell activation, which are critical for the development of cardiac fibrosis.

In vivo experiments showed that overexpression of LncRNA-IH in MI mice significantly increased the protein expression level of TGF-β1 in cardiac tissues compared with the MI group (Fig. 6D). ZEB1, a hallmark downstream target protein of the TGF-β1 signaling pathway, was also upregulated at both the protein (Fig. 6E) and mRNA (Fig. 6F) levels in the MI-LncRNA-IH group, confirming that LncRNA-IH overexpression activates the TGF-β1 signaling pathway in the heart after MI.

ELISA detection of serum samples revealed that compared with the Sham group, the serum TGF-β1 level in the MI group was significantly increased, and this increase was further enhanced in the MI-LncRNA-IH group (Fig. 6G), which was consistent with the changes in cardiac tissue, suggesting that LncRNA-IH can promote the systemic activation of the TGF-β1 signaling pathway. In vitro, overexpression of LncRNA-IH in primary cardiac fibroblasts (MCF) and fibroblast cell line PA12 significantly upregulated the mRNA expression of ZEB1 (Fig. 6H), indicating that LncRNA-IH can directly activate the TGF-β1 signaling pathway in fibroblasts.

Taken together, these results suggest that LncRNA-IH may promote cardiac fibrosis by activating the TGF-β1 signaling pathway.

## Discussion

This study demonstrates that LncRNA-IH exacerbates post-MI cardiac fibrosis by promoting cardiac fibroblast proliferation, migration, and activation, at least partially via the TGF-β1/ZEB1 pathway. These findings advance our understanding of lncRNA-mediated fibrosis regulation and highlight LncRNA-IH as a potential therapeutic target.

### LncRNA-IH: a hypoxia-responsive driver of fibroblast activation

Our results show that CIH upregulates LncRNA-IH in cardiac fibroblasts, consistent with prior evidence that hypoxia modulates lncRNA expression to regulate cellular functions (Saigusa et al. 2023; Guo et al. 2022). CIH exacerbates post-MI fibrosis, and LncRNA-IH’s fibroblast-specific localization (co-staining with col1A2) suggests it directly mediates hypoxia-induced fibrotic responses (Ilieva et al. 2022; Wang et al. 2020; Zhang et al. 2020). This aligns with studies showing fibroblast-enriched lncRNAs (e.g., H19 and MALAT1) regulate fibrosis (Yang et al. 2018; Chen et al. 2024; Ilieva et al. 2022), but LncRNA-IH is distinct in its hypoxia-dependent induction—linking environmental stress (e.g., sleep apnea-related hypoxia) to fibroblast activation (Lelli et al. 2015).

Cardiac-specific LncRNA-IH overexpression aggravated post-MI cardiac dysfunction (reduced LVEF, enlarged ventricular cavities) and structural remodeling (increased HW/BW ratio), hallmarks of adverse remodeling (Nagaraju et al. 2017). This phenotype was associated with increased collagen deposition and upregulated fibrotic markers (α-SMA, Col1, Col3), confirming its profibrotic role. A potential limitation of the present study is that AAV9, which was used for in vivo overexpression of LncRNA-IH, is not fibroblast-specific and can infect both cardiomyocytes and cardiac fibroblasts, meaning the observed in vivo phenotype might be partially influenced by LncRNA-IH expression in cardiomyocytes. However, supplementary experiments comparing LncRNA-IH expression levels between the two cell types after AAV9 transfection revealed significantly higher expression in cardiac fibroblasts than in cardiomyocytes, providing robust evidence that cardiac fibroblasts play a dominant role in mediating the observed phenotypic effects. In cardiac fibrosis, mRNA transcription and protein translation of fibrosis-related genes are often regulated by multiple layers. It is possible that in the early stage of MI, α-SMA has been transcriptionally activated (thus increasing mRNA level), but its protein synthesis or stability is regulated by other factors (such as ubiquitination-mediated degradation), resulting in no significant change in protein level. The reversal of these effects by LncRNA-IH knockdown in vitro further supports specificity. Together, these data establish LncRNA-IH as a functional mediator of fibroblast-driven fibrosis.

### Transcriptomic insights: LncRNA-IH and pathway dysregulation

Transcriptomic analysis identified 24 LncRNA-IH-regulated genes co-dysregulated in MI, including *Spon2* and *Ctxn3*. *Spon2*, involved in ECM remodeling in other tissues, may enhance fibroblast migration (Zhang et al. 2018), while *Ctxn3* (linked to cell adhesion) could promote fibroblast activation. These genes, alongside others like *Bahcc1* and *Nkx2-1*, warrant further investigation as downstream effectors (Stratton and McKinsey 2016).

GO enrichment highlighted “cellular process” (encompassing fibroblast proliferation/migration) and “structural molecule activity” (linked to ECM deposition) as key pathways, consistent with LncRNA-IH’s pro-fibrotic role. KEGG analysis linked LncRNA-IH to TGF-β1 signaling, a well-established fibrosis driver (Li et al. 2013). We confirmed that LncRNA-IH upregulates TGF-β1 and its downstream target ZEB1 in vivo and in vitro. Notably, serum TGF-β1 elevation suggests LncRNA-IH may systemically amplify fibrosis. A limitation of the present study is that transcriptomic KEGG enrichment analysis did not directly identify significant enrichment of the TGF-β1 pathway itself but only its downstream or synergistic pathways (e.g., ECM-receptor interaction, Calcium signaling pathway), and other fibrosis-related pathways (such as Cell adhesion molecules) revealed by transcriptomic data— which may contribute to LncRNA-IH-regulated cardiac fibrosis through complementary mechanisms—were not further explored as the study focused on the TGF-β1/ZEB1 axis. These limitations underscore the need for future studies to clarify the direct molecular mechanism by which LncRNA-IH activates the TGF-β1 pathway and systematically investigate the functional roles of other enriched pathways and their crosstalk with the TGF-β1 pathway.

ZEB1, traditionally associated with cancer metastasis (Lamouille et al. 2014), has recently been linked to cardiac fibrosis (Cufi et al 2010; Yuan et al. 2020). Our finding that LncRNA-IH directly upregulates ZEB1 in fibroblasts identifies a novel regulatory axis: LncRNA-IH → TGF-β1 → ZEB1 → ECM synthesis, expanding ZEB1’s role beyond epithelial contexts. It is important to note that while ZEB1 and TGF-β1 were not among the 24 significantly altered downstream genes of LncRNA-IH identified by transcriptomic analysis, we prioritized these two molecules because the TGF-β1 pathway is a well-established core regulator of cardiac fibrosis and ZEB1, as a key downstream transcription factor of the TGF-β1 pathway, has a well-documented role in promoting fibroblast activation and extracellular matrix remodeling.

### Clinical implications and therapeutic potential

Post-MI fibrosis lacks targeted therapies, with current treatments focusing on symptom management (McMurray et al. 2014). LncRNA-IH’s fibroblast specificity and role in TGF-β1 activation make it an attractive target. Antisense oligonucleotides (ASOs) and siRNAs targeting non-coding RNAs exhibit therapeutic potential in fibrosis (Bennett and Swayze 2010). For example, siRNA delivery systems targeting fibrogenic miRNAs (e.g., miR-21) demonstrate efficacy in reducing collagen deposition (Thum et al. 2008). Similarly, LncRNA-IH inhibition could mitigate fibrosis without affecting other cell types.

CIH, common in sleep-disordered breathing, worsens post-MI outcomes (Cain et al. 2015). Our data suggest targeting LncRNA-IH may counteract CIH-induced fibrosis, offering a personalized approach for patients with comorbid hypoxia. Combining LncRNA-IH inhibition with existing therapies (e.g., ACE inhibitors or SGLT2 inhibitors) could synergistically preserve cardiac function (McMurray et al. 2019; Kobayashi et al. 2016).

### Limitations and future directions

This study has limitations. First, the mechanism by which LncRNA-IH activates TGF-β1 remains unclear—whether it interacts with TGF-β1 directly or via miRNAs (e.g., as a ceRNA) requires exploration. Second, we focused on ZEB1, but TGF-β1 has other targets (e.g., Smads) that may contribute. Third, in vivo experiments used overexpression; LncRNA-IH knockout models are needed to confirm physiological relevance.

Future work should investigate: (1) LncRNA-IH’s interaction with TGF-β1 pathway components via RNA pull-down/ChIP; (2) long-term effects of LncRNA-IH inhibition on post-MI prognosis in animal models; (3) clinical relevance of LncRNA-IH expression in human post-MI samples.

## Conclusions

Long non-coding RNA-IH (LncRNA-IH) promotes post-myocardial infarction (post-MI) cardiac fibrosis and activates the TGF-β1/ZEB1 pathway. This work identifies a novel regulatory axis (LncRNA-IH → TGF-β1 → ZEB1) and highlights LncRNA-IH as a potential therapeutic target to improve post-MI outcomes by inhibiting fibroblast activation.

## Materials and methods

### Animals and experimental groups

Male C57BL/6 mice (8–10 weeks old, 22–25 g) were purchased from Beijing SPF Biotechnology Co., Ltd., Beijing, China. All animal experiments were approved by the Institutional Animal Care and Use Committee of Capital Medical University and conducted in accordance with the National Institutes of Health Guide for the Care and Use of Laboratory Animals.

Mice were randomly divided into the following groups (*n* = 6–8 per group):

- Sham group: Mice underwent thoracotomy without left anterior descending (LAD) coronary artery ligation.
- MI group: Mice were subjected to myocardial infarction (MI) via LAD ligation.
- MI + CIH group: MI-induced mice were exposed to chronic intermittent hypoxia (CIH) after surgery.
- MI + OE-LncRNA-IH group: MI-induced mice were injected with an adeno-associated virus (AAV) for cardiac-specific overexpression of LncRNA-IH (AAV9-LncRNA-IH, Genomeditech Co., Ltd., Shanghai, China).
- MI + AAV-NC group: MI-induced mice were injected with a control AAV (AAV9-NC) as a negative control.

### Myocardial Infarction (MI) model establishment

MI was induced as previously described with minor modifications (Li et al. 2017). Briefly, mice were anesthetized with 2% isoflurane (in 95% O_2_ and 5% CO_2_) and mechanically ventilated. A left thoracotomy was performed to expose the heart, and the LAD coronary artery was ligated with a 7–0 silk suture 2–3 mm below the left atrial appendage. Successful MI was confirmed by regional cyanosis of the left ventricle. Sham-operated mice underwent thoracotomy without LAD ligation.

### Chronic Intermittent Hypoxia (CIH) exposure

CIH was applied using a hypoxia chamber starting 24 h after MI surgery. The CIH protocol consisted of alternating cycles of 5 min of hypoxia (5% O_2_) and 10 min of normoxia (21% O_2_) for 8 h/day (9:00 AM–5:00 PM) for 4 weeks. Normoxic controls were maintained in room air under the same conditions.

### AAV-mediated LncRNA-IH overexpression in vivo

Recombinant AAV9 vectors encoding LncRNA-IH (AAV9-LncRNA-IH) or a negative control sequence (AAV9-NC) were constructed by Genomeditech Co., Ltd., Shanghai, China. Two weeks after MI induction, mice were injected with 100 μL of AAV9-LncRNA-IH or AAV9-NC (1 × 10^11^ vg/mL) via tail vein. Cardiac tissues were collected 4 weeks post-injection to verify LncRNA-IH overexpression by quantitative real-time PCR (qPCR).

### Echocardiographic analysis

Cardiac function was evaluated using a high-resolution ultrasound system (Vevo 2100, Fujifilm VisualSonics, Toronto, Canada) at 4 weeks post-MI. Mice were anesthetized with 1.5% isoflurane, and M-mode echocardiography was performed at the mid-papillary level. Parameters measured included left ventricular ejection fraction (LVEF), fractional shortening (FS), left ventricular internal diameter at end-diastole (LVIDd), and left ventricular internal diameter at end-systole (LVIDs). All analyses were performed by a blinded observer.

### Histological staining and quantification

Mice were euthanized at 4 weeks post-MI, and hearts were harvested, rinsed in cold PBS, and fixed in 4% paraformaldehyde for 24 h. Fixed hearts were embedded in paraffin and sectioned into 5-μm slices.

#### Masson’s trichrome staining

The collagen deposition was assessed by Masson’s staining (*n* = 6/group). Fibrotic area (blue-stained region) was quantified using Image-Pro Plus 6.0 software (Media Cybernetics, Rockville, MD, USA) and expressed as a percentage of the total left ventricular area. For Masson’s trichrome, power analysis shows that the sample size of 6/group has a 91% ~ 99% power assuming a 5% significance level with a two-sided test.

#### Hematoxylin and Eosin (HE) staining

Sections were stained with HE to evaluate cardiac structural remodeling, including left ventricular cavity size and ventricular wall thickness.

### Cell isolation and culture

#### Primary Cardiac Fibroblasts (MCF)

Cardiac fibroblasts were isolated from 1–3-day-old C57BL/6 mouse pups as previously described (Li et al. 2017). Briefly, hearts were minced and digested with 0.1% collagenase II and 0.25% trypsin–EDTA at 37 °C. Cells were plated in Dulbecco’s Modified Eagle Medium supplemented with 10% fetal bovine serum (FBS) and 1% penicillin–streptomycin, and incubated at 37 °C in 5% CO_2_. Fibroblasts were purified by differential adhesion and used at passages 2–4.

#### PA12 cell line

The PA12 cardiac fibroblast cell line was cultured in DMEM with 10% FBS and 1% penicillin–streptomycin under the same conditions as primary fibroblasts.

For CIH treatment in vitro, MCF and PA12 cells were exposed to CIH (8% O_2_ for 5 min/21% O_2_ for 10 min) in a hypoxia incubator for 72 h. Normoxic controls were maintained in 21% O_2_.

### Cell transfection

#### Overexpression of LncRNA-IH

MCF and PA12 cells were transfected with a LncRNA-IH overexpression plasmid (pcDNA3.1-LncRNA-IH) or empty vector (pcDNA3.1-NC) using Lipofectamine® 3000 Transfection Kit ([Cat. No. L3000-015], Invitrogen by Thermo Fisher Scientific, Carlsbad, CA, USA) according to the manufacturer’s protocol.

#### Knockdown of LncRNA-IH

Small interfering RNAs (siRNAs) targeting LncRNA-IH (si-LncRNA-IH) and negative control siRNA (si-NC) were synthesized by Hesheng Bio, Shanghai, China. Cells were transfected with si-LncRNA-IH or si-NC using Hiperfect Transfection Reagent ([Cat. No. 301705], Qiagen, Hilden, Germany) at a final concentration of 50 nM.

LncRNA-IH expression was verified by qPCR at 48 h post-transfection.

### Cell proliferation assay

Cell proliferation was assessed using the 5-bromo-2’-deoxyuridine (BrdU) incorporation assay ([Cat. No. G4102-50T], Servicebio, Wuhan, China). Transfected MCF or PA12 cells were seeded in 96-well plates (5 × 10^3^ cells/well) and incubated with BrdU (10 μM) for 4 h. After fixation and permeabilization, cells were incubated with an anti-BrdU antibody ([Cat. No. GB12051-50], Servicebio, Wuhan, China) at 4 °C overnight, followed by a fluorescein-conjugated secondary antibody. BrdU-positive cells were counted under a fluorescence microscope (Olympus, Tokyo, Japan) in 5 random fields per well, and the proliferation rate was calculated as (BrdU-positive cells/total cells) × 100%.

### Cell migration assay

Cell migration was evaluated using Transwell chambers (8-μm pore size, [Cat. No.3422], Corning Incorporated, Corning, NY, USA). Transfected MCF or PA12 cells (1 × 10^5^ cells/well) were suspended in serum-free DMEM and added to the upper chamber; the lower chamber contained DMEM with 10% FBS as a chemoattractant. After 24 h of incubation, non-migrated cells in the upper chamber were removed with a cotton swab, and migrated cells on the lower membrane were fixed with 4% paraformaldehyde, stained with 0.1% crystal violet, and counted under a light microscope (5 random fields per well).

### Quantitative Real-Time PCR (qPCR)

Total RNA was extracted from cardiac tissues or cells using TRIzol reagent ([Cat. No. 15596026], Ambion by Life Technologies, Carlsbad, CA, USA) and reverse-transcribed into cDNA using RevertAid First Strand cDNA Synthesis Kit ([Cat. No. K1622], Thermo Scientific, Waltham, MA, USA). qPCR was performed using SYBR Premix Ex Taq™ ([Cat. No. RR420A], Takara) on a StepOnePlus Real-Time PCR System (Thermo Fisher Scientific). The relative expression of target genes was calculated using the 2^-^ΔΔCt method, with GAPDH as the internal reference.

Primer sequences are listed in Table 1:

**Table 1:** Primer sequences of target genes used in the study

Target Gene	Forward Primer (5’ → 3’)	Reverse Primer (5’ → 3’)
LncRNA-IH	CCCAGACTTGAGTTGCGAGT	TCCACTCCAGGACCCAAGAA
α-SMA	GTACCACCATGTACCCAGGC	GAAGGTAGACAGCGAAGCCA
Col1a1	AAGAAGCACGTCTGGTTTGGAG	GGTCCATGTAGGCTACGCTGTT
Col3	GTGGCAATGTAAAGAAGTCTCTGAAG	GGGTGCGATATCTATGATGGGTAG
ZEB1	ATTACCAGGAGGCAGTGACAGG	CCACATTCGGATCATGGTTTTG
GAPDH	CCTCGTCCCGTAGACAAAATG	TGAGGTCAATGAAGGGGTCGT

### Western blot analysis

Total protein was extracted from cardiac tissues or cells using RIPA lysis buffer ([Cat. No. G2002-100ML], Servicebio, Wuhan, China) containing protease and phosphatase inhibitors ([Cat. No. G2007-1ML], Servicebio, Wuhan, China). Protein concentration was determined using a BCA Protein Assay Kit ([Cat. No. G2026-200T], Servicebio, Wuhan, China). Equal amounts of protein (50 μg) were separated by 10% SDS-PAGE and transferred to PVDF membranes (Cat. No. WGPVDF45, Servicebio, Wuhan, China). Membranes were blocked with 5% non-fat milk in TBST for 1 h at room temperature, then incubated with primary antibodies overnight at 4 °C:

- Anti-α-SMA ([Cat. No. GB111364], Servicebio, Wuhan, China, 1:1000)
- Anti-Col1a1([Cat. No. GB115707], Servicebio, Wuhan, China, 1:1000)
- Anti-Col3 ([Cat. No. GB111629], Servicebio, Wuhan, China, 1:1000)
- Anti-TGF-β1 ([Cat. No. GB15179], Servicebio, Wuhan, China, 1:1000)
- Anti-ZEB1 ([Cat. No. ab155249], Abcam, Cambridge, UK, 1:1000)
- Anti-GAPDH ([Cat. No. GB15004], Servicebio, Wuhan, China, 1:5000, internal control)

Membranes were then incubated with HRP-conjugated secondary antibodies ([Cat. No. GB23303], Servicebio, Wuhan, China, 1:5000) for 1 h at room temperature. Protein bands were visualized using an enhanced chemiluminescence (ECL) kit ([Cat. No. G2020-25ML], Servicebio, Wuhan, China) and quantified using ImageJ software (NIH, Bethesda, MD, USA).

### Fluorescence In Situ Hybridization (FISH)

FISH was performed to localize LncRNA-IH in cardiac tissues using a LncRNA-IH-specific probe labeled with Cy3. Paraffin sections were deparaffinized, rehydrated, and subjected to antigen retrieval. After prehybridization, sections were incubated with the probe (100 nM) at 37 °C overnight. For co-localization with cardiac fibroblasts, sections were stained with anti-α-SMA (Cat. No. GB111364-100, Servicebio, Wuhan, China, 1:500)、anti-CTNT (Cat. No. GB113806-100, Servicebio, Wuhan, China, 1:500)、anti-CD68 antibody (Cat. No. GB113109-100, Servicebio, Wuhan, China, 1:500) and anti-VWF antibody (Cat. No. GB11020-100, Servicebio, Wuhan, China, 1:500) followed by a FITC-conjugated secondary antibody. Nuclei were counterstained with DAPI. Images were captured using a confocal microscope (Zeiss, Oberkochen, Germany).

### Enzyme-Linked Immunosorbent Assay (ELISA)

Serum TGF-β1 levels were measured using a mouse TGF-β1 ELISA Kit ([Cat. No. 88–8350], Invitrogen, Carlsbad, California, USA) according to the manufacturer’s instructions. Briefly, serum samples (100 μL) were added to pre-coated wells and incubated at 37 °C for 2 h. After washing, biotinylated detection antibody was added, followed by streptavidin-HRP. The reaction was developed with TMB substrate, and absorbance was measured at 450 nm using a microplate reader (Bio-Rad, Hercules, CA, USA). TGF-β1 concentration was calculated from a standard curve.

### Transcriptomic profiling

Total RNA was extracted from cardiac tissues of Sham, MI, and MI + OE-LncRNA-IH groups (*n* = 3 per group) and subjected to RNA sequencing by Metware Biotechnology Co., Ltd., Wuhan, China. Libraries were constructed using a VAHTS Universal V10 RNA-seq Library Prep Kit (Premixed version) ([Cat. No. NR616-02], Vazyme Biotech Co., Ltd., Nanjing, China) and sequenced on an Illumina NovaSeq X Plus System (150-bp paired-end reads).

Raw reads were filtered to remove low-quality reads and adapter sequences. Clean reads were mapped to the mouse genome (mm10) using Hisat2. Differentially expressed genes (DEGs) were identified using DESeq2 with thresholds of |log_2_(fold change)|> 1 and adjusted *p* < 0.05. Functional enrichment analysis (GO and KEGG) was performed using clusterProfiler in R.

### Statistical analysis

All data are presented as mean ± standard error of the mean (SEM). Statistical analyses were performed using GraphPad Prism 9.0 (GraphPad Software, San Diego, CA, USA) and SPSS 22.0 (IBM, Armonk, NY, USA). Differences between multiple groups were analyzed by one-way analysis of variance (ANOVA) followed by Tukey’s post-hoc test. A *p*-value < 0.05 was considered statistically significant.

## Supplementary Information

Supplementary Material 1. Supplementary figures. Fig. S1. Chronic intermittent hypoxia promotes the expression of LncRNA-IH in cardiac fibroblasts. Fig. S2. The Localization of LncRNA-IH in Cardiac Endothelial Cells and Macrophages. Fig. S3. LncRNA-IH promotes the proliferation and migration of cardiac fibroblasts. Fig. S4. LncRNA-IH promotes the proliferation and migration of cardiac fibroblasts. Fig. S5. GO functional annotation analysis of LncRNA-IH-associated mRNAs after MI. Fig. S6. Gene Set Enrichment Analysis (GSEA) of GO functions and KEGG pathways for LncRNA-IH-associated mRNAs in different comparison groups.

## Data Availability

All raw data (e.g., qPCR raw Ct values, Western blot original images, histological staining quantification data) and analysis code used in this study are available from the corresponding author (Chaowei Hu: chaowei_hu@126.com; Yanwen Qin: qinyanwen@ccmu.edu.cn) on reasonable request. The RNA-seq dataset generated in this study has been deposited in the National Center for Biotechnology Information (NCBI) database: PRJNA1298696.

## Abbreviations

MI: Myocardial Infarction
CIH: Chronic Intermittent Hypoxia
lncRNA: Long Non-Coding RNA
LncRNA-IH: LncRNA nonnmmut065573 (tentatively designated)
ECM: Extracellular Matrix
AAV: Adeno-Associated Virus
AAV9-NC: AAV9 Negative Control
OE-LncRNA-IH: LncRNA-IH Overexpression
LAD: Left Anterior Descending (Coronary Artery)
qPCR: Quantitative Real-Time Polymerase Chain Reaction
FISH: Fluorescence In Situ Hybridization
BrdU: 5-Bromo-2’-Deoxyuridine
ELISA: Enzyme-Linked Immunosorbent Assay
DEGs: Differentially Expressed Genes
GO: Gene Ontology
KEGG: Kyoto Encyclopedia of Genes and Genomes
GSEA: Gene Set Enrichment Analysis
α-SMA: α-Smooth Muscle Actin
Col1: Collagen Type I
Col3: Collagen Type III
LVEF: Left Ventricular Ejection Fraction
FS: Fractional Shortening
LVIDd: Left Ventricular Internal Diameter at End-Diastole
LVIDs: Left Ventricular Internal Diameter at End-Systole
HW/BW: Heart Weight-to-Body Weight Ratio
SD: Standard Deviation
SEM: Standard Error of the Mean
ANOVA: Analysis of Variance
MCF: Primary Cardiac Fibroblasts
RIPA: Radio-Immunoprecipitation Assay
BCA: Bicinchoninic Acid
SDS-PAGE: Sodium Dodecyl Sulfate–Polyacrylamide Gel Electrophoresis
PVDF: Polyvinylidene Fluoride
ECL: Enhanced Chemiluminescence
