# Supplementary material for: LncRNA nonnmmut065573 promotes post-myocardial infarction cardiac fibrosis and activates the TGF-β1/ZEB1 pathway
Source: Cell Regen. 2026 Jan 21;15:5. doi: 10.1186/s13619-025-00275-5 (PMC12824076; doi:10.1186/s13619-025-00275-5)
Supplement: Supplementary file 1 — Supplementary Material 1. Supplementary figures. Fig. S1. Chronic intermittent hypoxia promotes the expression of LncRNA-IH in cardiac fibroblasts. Fig. S2. The Localization of LncRNA-IH in Cardiac Endothelial Cells and Macrophages. Fig. S3. LncRNA-IH promotes the proliferation and migration of cardiac fibroblasts. Fig. S4. LncRNA-IH promotes the proliferation and migration of cardiac fibroblasts. Fig. S5. GO functional annotation analysis of LncRNA-IH-associated mRNAs after MI. Fig. S6. Gene Set Enrichment Analysis (GSEA) of GO functions and KEGG pathways for LncRNA-IH-associated mRNAs in different comparison groups. [file 13619_2025_275_MOESM1_ESM.docx]

Title：**LncRNA** **nonnmmut065573 Promotes Post-Myocardial Infarction Cardiac Fibrosis and Activates the TGF-β1/ZEB1 Pathway**

Running Title：LncRNA nonnmmut065573 Promotes Post-MI Cardiac Fibrosis

Author List: Chaowei Hu^1^*,Lijie Han^1^, Zhiyong Du^1^, Huahui Yu^1^, Yunhui Du^1^, Linyi Li^1^, Haili Sun^1^,Yu Wang^1^, Xiaoqian Gao^1^, Xuechun Sun^1^, Zihan Zhang^1^ ,Lanqing Liu^1^ , Yanjing Zhang^1^ and Yanwen Qin ^1^*.

1. Key Laboratory of Remodeling-related Cardiovascular Diseases, Beijing Anzhen Hospital, Capital Medical University, Beijing Institute of Heart, Lung and Blood Vessel Diseases, Beijing 100029, China.

*Correspondence: chaowei_hu@126.com（Chaowei Hu），qinyanwen@ccmu.edu.cn; qinyanwen@vip.126.com (Yanwen Qin)

**Supplementary figures 1-6**


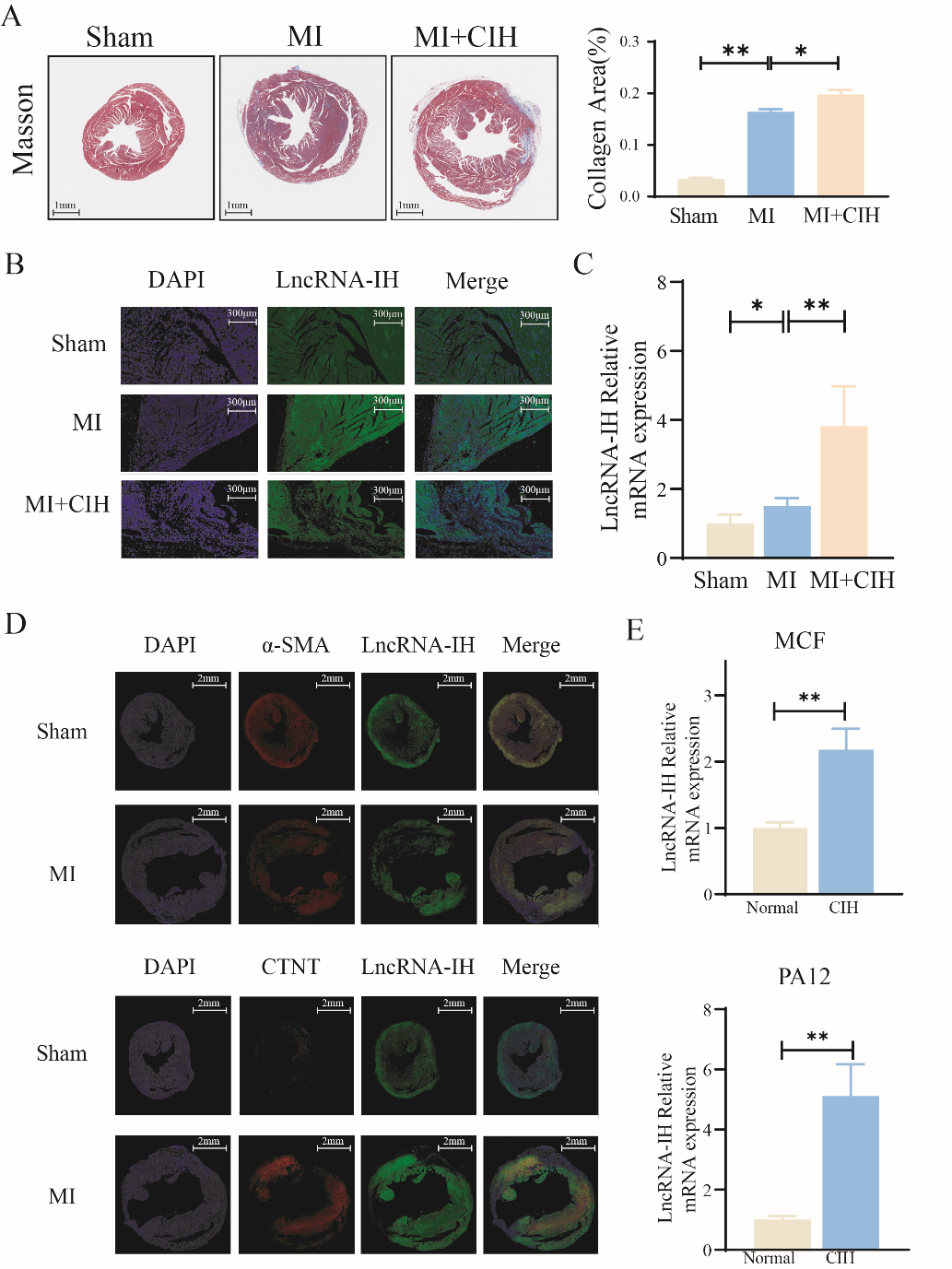


**Fig. S1.** **Chronic intermittent hypoxia promotes the expression of LncRNA-IH in cardiac fibroblasts.** (A)Cross sections of heart tissues were stained by Masson, and cardiac fibrosis were determined. n = 6 for each group. (B) Representative images of FISH of LncRNA-IH in Cardiac tissue co-labeled with DAPI (blue). (C) LncRNA-IH expression profiles were detected by qRT-PCR in different groups. (D) Representative images of fluorescent in situ hybridizations (FISH) of LncRNA-IH co-labeled with DAPI (blue) and α-SMA (myofibroblasts) (red) or CTNT (cardiomyocytes) (red) to observe the localization of LncRNA-IH in cardiac cells. (E) The mRNA expression levels of LncRNA-IH were measured in both MCF and PA12 cell lines after CIH treatment.n = 8 for Sham, n = 6 for MI and n = 8 for MI + CIH. ^*^P < .05, ^**^P < .01. Data are presented as mean ± SEM.MI, myocardial infarction; MI+CIH, After myocardial infarction (MI), chronic intermittent hypoxia treatment was administered after myocardial infarction.


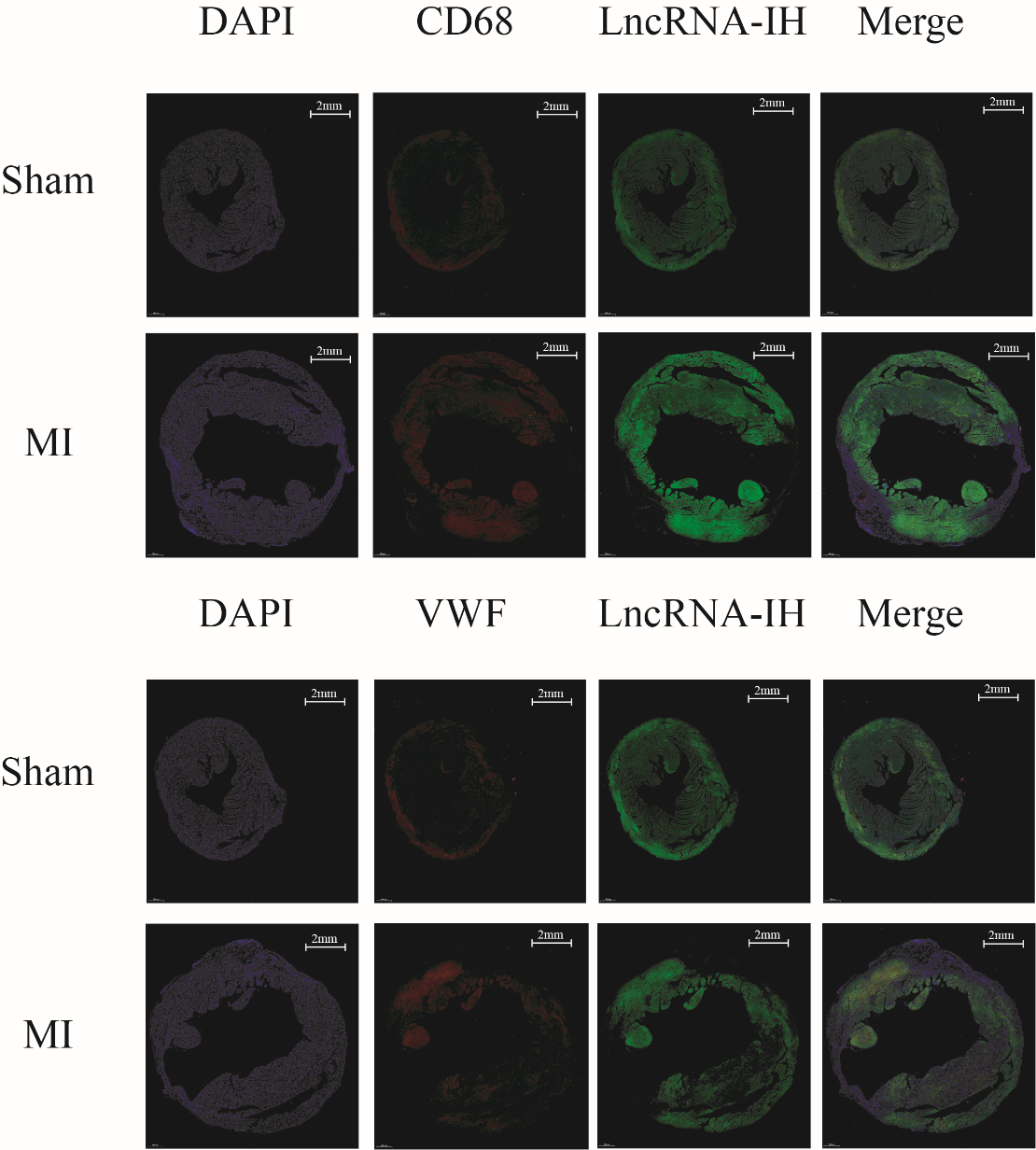


**Fig. S2**. **The Localization of LncRNA-IH in Cardiac Endothelial Cells and Macrophages.** Eresentative images of fluorescent in situ hybridizations (FISH) of LncRNA-IH co-labeled with DAPI (blue) and vWF (endothelial cells) (red) or CD68 (macrophages) (red) to observe the localization of LncRNA-IH in cardiac cells.


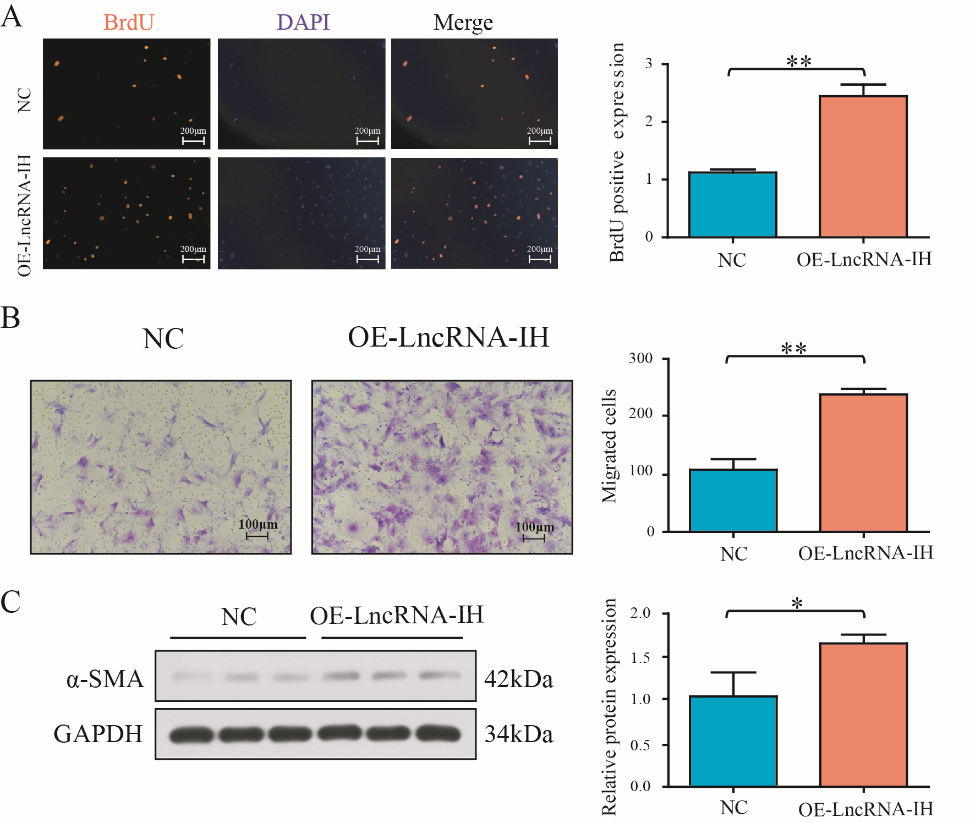


**Fig. S3.** **LncRNA-IH promotes the proliferation and migration of cardiac fibroblasts.** (A) Quantitative analysis of the percentage of BrdU-positive cells in each group was performed, and the data are presented as Mean±SEM, with n=3/group. One-way analysis of variance (One-way ANOVA) and Tukey’s multiple comparison test were used. ^*^P<0.05 and ^**^P<0.01 vs. the NC group. (B) Transwell assay to determine the effect of LncRNA-IH on the migration of cardiac fibroblasts. data are presented as Mean±SEM, with n=3/group. *P<0.05 and **P<0.01 vs. the NC group. (C) Level of α-SMA in cardiac fibroblasts was detected by Western blotting. The relative intensities are shown in the panel. GAPDH served as a loading control. n=3 per group.Data are presented as *P < 0.05, **P < 0.01. Data are presented as mean ± SEM.


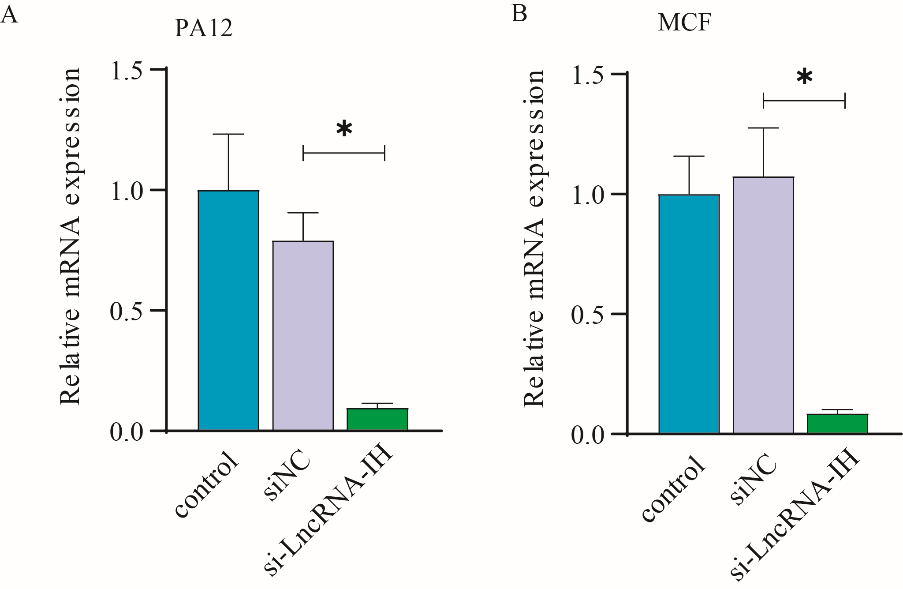


**Fig. S4. LncRNA-IH promotes the proliferation and migration of cardiac fibroblasts.** The expression of LncRNA-IH was validated by qPCR analysis in control, siNC and OE- LncRNA-IH groups in PA12（A）and MCF（B）cells, respectively. n = 3/group. *P < .05. Data are presented as mean ± SEM.


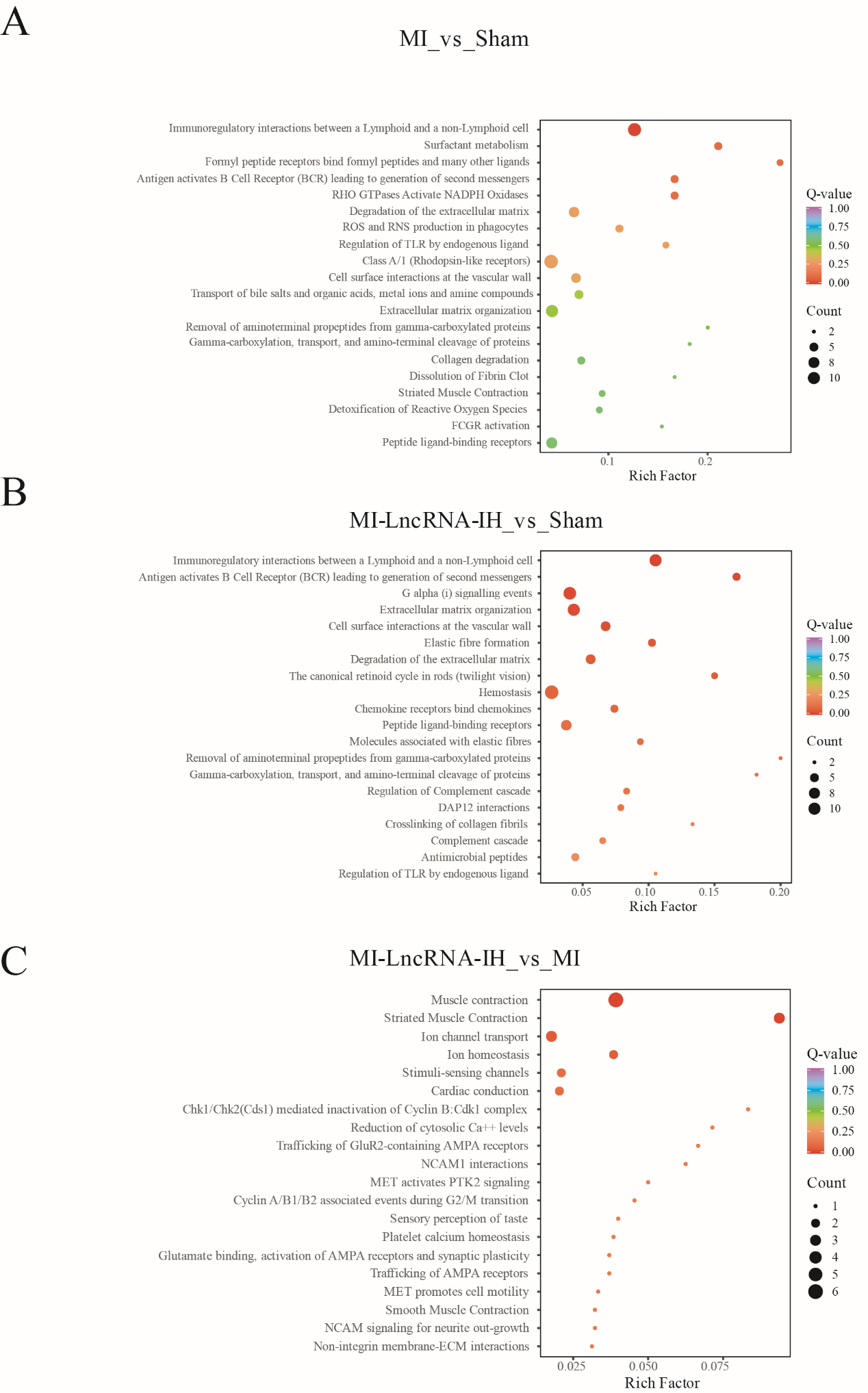


**Fig. S5. GO functional annotation analysis of LncRNA-IH-associated mRNAs after MI.** GO enrichment dot plots of differentially expressed genes show the significantly enriched GO terms in MI vs Sham, MI-LncRNA-IH vs MI and MI-LncRNA-IH vs Sham groups. The size of bubbles represents the number of genes in each term (Count), the color indicates the Q-value (darker color means smaller Q-value and more significant enrichment), and the x-axis denotes the rich factor (reflecting the enrichment degree of genes in the term).


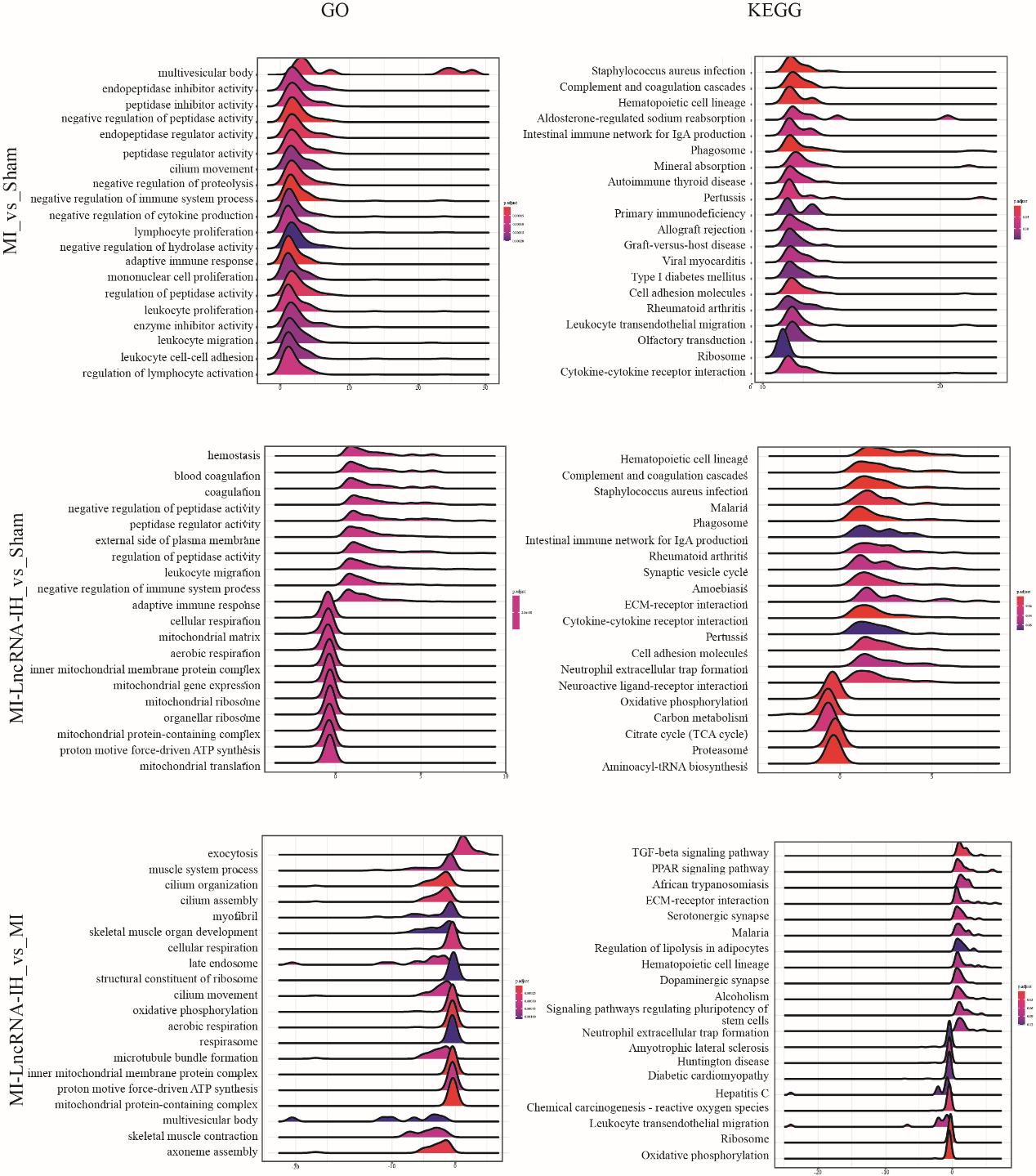


**Fig. S6. Gene Set Enrichment Analysis (GSEA) of GO functions and KEGG pathways for LncRNA-IH-associated mRNAs in different comparison groups.** GSEA was performed to identify significantly enriched biological processes and pathways among LncRNA-IH-associated mRNAs in three comparison groups: MI-LncRNA-IH vs Sham, MI vs Sham, and MI-LncRNA-IH vs MI. Enriched terms include GO functions such as endopeptidase regulator activity, negative regulation of immune system process, and leukocyte proliferation, as well as KEGG pathways including Cell adhesion molecules, Leukocyte transendothelial migration, and Oxidative phosphorylation. These results reflect the potential biological functions and pathway associations of LncRNA-IH-related mRNAs in the context of myocardial infarction.
